# Paediatric Emergency Department Mental Health and Behavioural Presentations in Australia Before and After the Onset of the COVID‐19 Pandemic: Retrospective Observational Study

**DOI:** 10.1111/jpc.70046

**Published:** 2025-03-31

**Authors:** Jackson Newberry‐Dupe, Glenn Melvin, Kylie King, Marietta John White, Vicki Anderson, Franz E. Babl, Meredith L. Borland, Stuart R. Dalziel, Harriet Hiscock, Bruce J. Tonge, Paul Buntine, Brooke Charters, Megan Hamilton, Amit Kochar, Alastair Meyer, Viet Tran, Emogene Aldridge, Giles Barrington, Liam Hackett, Nicole Lowry, Joseph Miller, Sebastian Wrobel, Rohan Borschmann, Simon Craig

**Affiliations:** ^1^ Centre for Mental Health and Community Wellbeing, Melbourne School of Population and Global Health University of Melbourne Parkville Australia; ^2^ Centre for Adolescent Health Murdoch Children's Research Institute Parkville Victoria Australia; ^3^ School of Psychology Deakin University Melbourne Victoria Australia; ^4^ Turner Institute for Brain and Mental Health Monash University Melbourne Victoria Australia; ^5^ Paediatric Emergency Department, Monash Medical Centre, Emergency Service Monash Health Clayton Victoria Australia; ^6^ Department of Paediatrics, School of Clinical Sciences Monash University Clayton Victoria Australia; ^7^ Department of Paediatrics University of Melbourne Parkville Australia; ^8^ Clinical Sciences Research Murdoch Children's Research Institute Parkville Victoria Australia; ^9^ Emergency Research Group Murdoch Children's Research Institute Parkville Victoria Australia; ^10^ Department of Critical Care University of Melbourne Parkville Australia; ^11^ Department of Emergency Medicine Royal Children's Hospital Parkville Australia; ^12^ Paediatric Emergency Department Perth Children's Hospital Nedlands Western Australia Australia; ^13^ School of Medicine, Divisions of Emergency Medicine and Paediatrics University of Western Australia Perth Western Australia Australia; ^14^ Starship Children's Hospital Auckland New Zealand; ^15^ University of Auckland Auckland New Zealand; ^16^ Health Services Murdoch Children's Research Institute Parkville Victoria Australia; ^17^ Centre for Developmental Psychiatry and Psychology Monash University Clayton Victoria Australia; ^18^ Emergency Medicine Research Program Eastern Health Box Hill Australia; ^19^ Eastern Health Clinical School Monash University Clayton Victoria Australia; ^20^ Department of Emergency Medicine Gold Coast University Hospital Southport Queensland Australia; ^21^ Emergency Department University Hospital Geelong Geelong Victoria Australia; ^22^ Emergency Consultant Women's and Children's Hospital Adelaide Australia; ^23^ Department of Acute Care Medicine University of Adelaide Adelaide Australia; ^24^ Emergency Department, Casey Hospital Monash Health Clayton Victoria Australia; ^25^ Royal Hobart Hospital Tasmanian Health Service Hobart Australia; ^26^ Tasmanian School of Medicine University of Tasmania Hobart Australia; ^27^ Department of Emergency Medicine, Box Hill Hospital Eastern Health Melbourne Australia; ^28^ Department of Psychiatry, Warneford Hospital University of Oxford Oxford UK; ^29^ Justice Health Group, Faculty of Health Sciences Curtin University Perth Australia

**Keywords:** adolescent, Australia, COVID‐19, emergency department, lockdown, mental health, paediatric

## Abstract

**Objectives:**

Although child and adolescent mental health and behavioural presentations to hospital emergency departments (EDs) increased during the first 2 years of the COVID‐19 pandemic (2020 and 2021), little is known about the characteristics of these presentations. We aimed to compare demographic, clinical and psychosocial profiles of paediatric presentations to Australian EDs before and after the onset of the pandemic.

**Methods:**

We conducted a retrospective observational study of 100 randomly sampled presentations by children (6–11‐year‐olds) and adolescents (12–17‐year‐olds) to 10 Australian EDs between 1 January and 31 December 2019 (pre‐COVID‐19) and 1 January and 31 December 2021 (COVID‐19). Using a multilevel factor model, we compared the pre‐COVID‐19 and COVID‐19 cohorts regarding demographic characteristics, diagnoses, precipitants, time‐to‐treatment, length of stay, and discharge disposition.

**Results:**

The COVID‐19 period was characterised by increased presentations by adolescents and girls. Compared to the pre‐COVID‐19 cohort, the COVID‐19 cohort experienced increased median waiting times (48 and 72 min, respectively), median length of ED stay (4.7 and 5.4 h), and likelihood of admissions to the ED short stay unit (9.5% and 12.9%). Patients in the COVID‐19 cohort were more likely to present with self‐harm and suicidal thoughts/behaviours, eating disorders, neurodevelopmental and neurocognitive disorders, and psychosocial stressors, and less likely to have diagnoses of disruptive behaviour, impulse control, and conduct disorders.

**Conclusions:**

Young people presenting to the ED in 2021 for mental health reasons were more likely to wait longer, stay longer, have a diagnosis of intentional self‐harm and/or a neurodevelopmental disorder, and report psychosocial stressors.

## 1 Introduction

Australia's response to the coronavirus pandemic (COVID‐19)—in terms of disease containment, reducing COVID‐19‐related deaths, and cost‐effectiveness of government interventions—has been described as world‐leading [1]. From January 2020 to October 2021, Australia maintained a ‘zero‐COVID’ policy to manage the pandemic. From 11 October 2021, with vaccine targets largely met, a transition to ‘living with COVID’ was made and the national state of emergency was lifted [2]. However, the zero‐COVID experience was not uniform across Australia. States closed borders and implemented state‐level restrictions during local infection waves, with restrictions in the state of Victoria reported as the longest and strictest in Australia [3]. Paediatric COVID‐19 epidemiology was highly variable across Australian states [4].

The impacts of multiple lockdowns, a prolonged state of emergency, and polarising media and public discourse [4, 5] were associated with community distress [6]. Internationally, studies reported increased prevalence of anxiety and mood disorders after the onset of the pandemic [7]. In Australian states and cities where COVID‐19 lockdowns occurred, children and adolescents experienced higher levels of disruption to their social, educational and domestic routines than other age groups [8, 9]. This resulted in increased loneliness, distress, and social vulnerabilities, with the potential for significant disruption to developmental trajectories [9]. During infection waves, public hospitals suspended non‐urgent elective surgeries and saw increased respiratory presentations alongside reductions in most other types of illness [10], except for mental health [11]. Emergency departments (EDs) across Australia reported increased presentations by children and adolescents for mental health issues and self‐harm (including suicidal ideation and behaviour) over 2020, 2021, and 2022 [11, 12, 13, 14]. The clinical and psychosocial profiles of these children and adolescents remain under‐investigated.

The aim of this study was to compare the profiles of children and adolescents presenting to hospital EDs in Australia for mental health‐related reasons before the onset of the COVID‐19 pandemic in 2019 with those in the second year of the pandemic (2021).

## 2 Method

### 2.1 Study Design and Setting

A multi‐centre retrospective cohort study of school‐aged children and adolescents (6–17‐years old) attending Australian EDs with a primarily mental health‐related presentation was conducted. Data were obtained from 10 hospitals in three states: eight metropolitan Victorian hospitals (two paediatric tertiary referral, one mixed adult/paediatric tertiary referral, and five mixed non‐tertiary), one regional mixed adult/paediatric tertiary hospital in Tasmania, and one metropolitan mixed non‐tertiary hospital in Queensland. Hospitals were recruited via an invitation to participate, distributed to 30 sites within the Paediatric Research in Emergency Departments International Collaborative (PREDICT) Network [15], a collective of paediatric EDs across Australia and New Zealand. We examined presentations between 1 January and 31 December 2019 (pre‐COVID‐19 group) and between 1 January and 31 December 2021 (COVID‐19 group). Ethical approval was granted by the Monash Health Human Research Ethics Committee (RES‐19‐0000‐896A).

### 2.2 Participants

Eligible participants were identified by searching the local ED Information System (EDIS) for all patients aged 6–17 years who attended the ED within the pre‐COVID‐19 or COVID‐19 period with a primarily mental health‐related presentation. Due to the different drivers, symptoms, and diagnostic methodologies of mental health in children under 6 years old [11, 14, 16], presentations by this age group were excluded from the current analysis. Presentations were eligible if the patient was: (a) referred to the mental health team from the ED; and/or (b) discharged with a mental health diagnosis (defined according to the International Statistical Classification of Diseases and Related Health Problems, Tenth Revision, Australian Modification [ICD‐10‐AM] codes relating to mental and behavioural disorders [F01–F99], emotional state, appearance and behaviour [R45.0–R46.8]); and/or (c) a diagnosis consistent with intentional self‐harm (X71–X83). Presentations were excluded if the only presenting complaint was intoxication with drugs or alcohol (Y91.0–Y91.9; excludes poisoning: T519). Table S1 contains a full list of included diagnoses.

### 2.3 Diagnostic Classification

Within Australian EDs, patients who present for mental health reasons are reviewed by ED clinicians and/or mental health teams, who categorise the presenting complaint using ICD‐10‐AM diagnostic codes to determine the most appropriate treatment/referral options [17]. These codes are numerous, highly detailed, and precise, allowing for rapid categorisation of patients within the busy ED setting. Codes used in the ED typically capture the presenting behaviours (e.g., destructive behaviours, drug ingestion) and new or existing mental health diagnoses (e.g., anorexia nervosa, borderline personality disorder). Discharge diagnoses were identified in discharge notes or, where these were incomplete, within mental health assessment notes. Given the precise detail of ICD‐10‐AM codes, diagnoses were grouped within the major categories of the Diagnostic and Statistical Manual of Mental Disorders, fifth edition (DSM‐5) (Table S2). Diagnostic impressions documented in the ED may not result from an extensive assessment as would typically occur in an inpatient, outpatient, or community setting [15]. Therefore, diagnostic categories described in this study may reflect preliminary impressions and should be interpreted as an indication of the behaviours and personality structures patients presented with and the likely treatment and referral pathways indicated for them within current practice guidelines, rather than a conclusive diagnosis.

### 2.4 Data Collection

At each hospital site, all eligible mental health presentations from 2019 and 2021 were exported from the EDIS into two excel spreadsheets (one for each year). All presentations, including repeat presentations, were included in the list, which was randomised using the ‘RAND’ function in excel. At each site, researchers were instructed to select up to 100 unique patient presentations per year (200 across both years) sequentially from their randomised list. It was decided a priori that 100 presentations from each period would be feasible and adequately representative of overall ED mental health visits. Where a patient presented multiple times, only the presentation closest to the top of the randomised list was selected, with any additional presentations lower down the list excluded (regardless of which presentation occurred first in the calendar year). For one site, less than 100 eligible presentations occurred, so randomisation of the list was not necessary (Table S3).

Medical record data were extracted for each randomly selected presentation from the computerised EDIS by trained research staff (abstractors), in line with recommended chart review methods [18]. Abstractors were given explanatory notes and met J.N.D. and M.J.W. regularly to resolve uncertainty. Data were de‐identified locally and entered into a password‐protected, central web‐based database (REDCap; Research Electronic Data Capture, hosted at Monash University [19, 20]). Abstracted data included routinely collected administrative and clinical (e.g., diagnosis/es, precipitants, history of mental illness, and social background) information. Although not routinely collected, gender and/or sexual diversity (GSD) was extracted from medical notes where available. Gender was defined as girl, boy, or non‐binary, according to the recorded gender/sex of patients in the medical notes. Transgender young people were categorised according to their preferred gender. If patient sex was described as female or male using the binary coding available in the computerised EDIS, with no mention of gender identity in other notes, this was assumed to align with preferred gender. The collection instrument included pre‐defined multiple‐choice options, with some opportunities for free text description. Free text data were coded using directed content analysis, first attempting to reintegrate them into the pre‐defined categories, with new categories created only if they could not be reintegrated. Coding was conducted by J.N.D., with 20% dual coded by E.A. for consistency. Discrepancies were resolved through discussion with R.B., S.C., K.K., and G.M.

### 2.5 Urgency

The urgency of ED presentations was defined using the five‐level Australasian Triage Scale (ATS; see Table S4). Each presentation was classified into a high urgency (ATS category l or 2; i.e., needing to be seen immediately or within 10 min, respectively) or lower urgency episode (ATS categories 3–5; i.e., needing to be seen within 30–120 min).

### 2.6 Psychosocial Factors

Psychosocial data were extracted from patient assessment records and grouped into broad categories: family/personal history of mental illness; social issues/vulnerabilities; housing and economic factors; trauma, abuse and neglect; relational problems (with parents, partners, and peers); substance use; medication changes; living situation; and other life stressors (including school‐related stressors).

### 2.7 Statistical Analysis

Data were analysed in Stata IC (V16.1, StataCorp, College Station, Texas, USA). Outliers were identified and either corrected or left untouched, based on site feedback and review of available data.

Descriptive statistics were generated to describe the participant profile. Categorical data were described as counts and percentages, while continuous data were described with medians and interquartile ranges. To account for clustering within hospitals, comparisons for binary categorical variables were made using the Mantel–Haenszel *Χ* ^2^ test. For variables with three or more groups, categories were compared to a single reference group [21]. Due to the skewed nature of hospital time and date data [22], a log transformation of continuous variables was conducted. Continuous data comparisons were made using a linear mixed model with a fixed effect of year and a random effect of hospital, following the restricted maximum likelihood (REML) method to account for clustering within a multilevel exploratory design [21]. Coefficients were exponentiated to report geometric means and confidence intervals (CIs).

## 3 Results

### 3.1 Demographic and Clinical Characteristics

**FIGURE 1:**
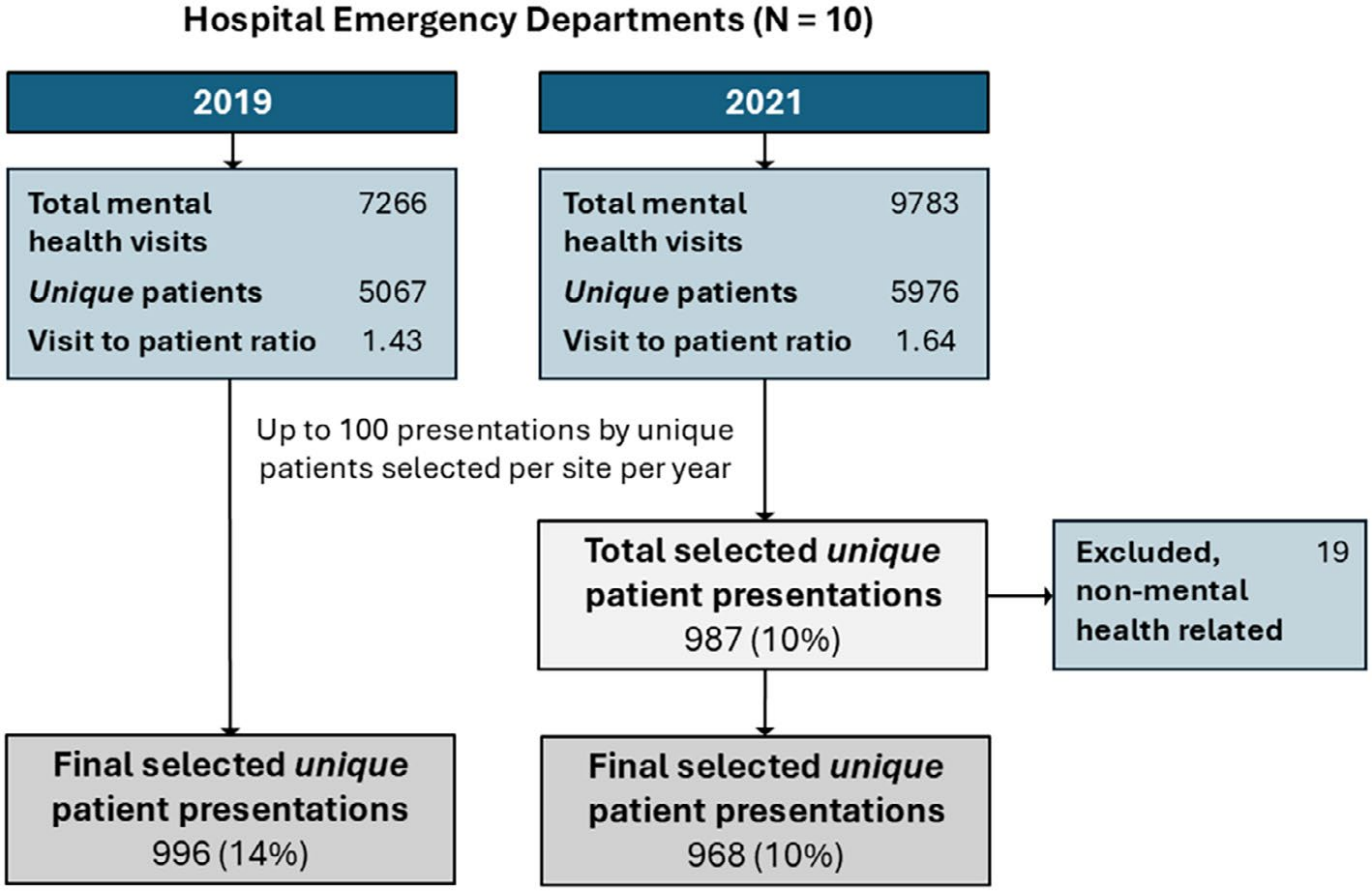
Patient recruitment flow diagram.

Figure 1 provides a flow diagram of patient recruitment. Across 10 sites, 996 ED records were included from 2019 and 968 were included from 2021.

One site had fewer than 100 eligible presentations, and some larger sites inadvertently collected over 100 from their randomised list of eligible presentations (20 additional presentations across five sites, mean of 3.33 per site [range of 1–7]; see also, Table S3). Since the analysis does not require an equal number of cases from each site [21] and removal of these cases would reduce power without decreasing risk of bias, they were retained. Following sample selection from randomised lists at each site, 19 patients selected at a single hospital network (*n* = 3 sites) from 2021 (2% of 2021 total across sites) were identified as acute alcohol intoxication presentations with no mental health‐related concerns—review of records confirmed ineligibility and they were removed prior to analysis. As data collection had concluded, it was not possible to replace removed patients. As such, site totals ranged from 82 to 107 in 2019 and 68 to 106 in 2021. In both 2019 and 2021, the majority of those presenting were girls (63.4% and 68.0%, respectively), were aged 12–17 years (89.7% vs. 92.8%, respectively), and were born in Australia (Table 1). Due to the low number of non‐binary young people (*N* = 16), gender is only reported for boys and girls. There was a 157% increase in reporting of gender and/or sexual diversity amongst young people presenting in 2021, compared to 2019 (95% CI [62%, 401%]).

**TABLE 1:** Comparison of demographic, service use, and discharge disposition characteristics of child and adolescent mental health ED presentations in 2019 and 2021.

	2019 (*N* = 996)	2021 (*N* = 968)	RR (95% CI)
Demographic profile			
Median age in years (IQR)	15.0 (14.0–16.0)	15.0 (14.0–16.0)	
Age group (years)^a^			
Children (6–11)	103 (10.3%)	70 (7.2%)	Reference
Adolescent (12–17)	893 (89.7%)	898 (92.8%)	**1.04 (1.01–1.06)**
Gender			
Boys^b^	359 (36.1%)	290 (30.4%)	Reference
Girls^b^	635 (63.9%)	664 (69.6%)	**1.07 (1.01–1.14)**
Gender and sexual diversity^c^	24 (2.4%)	60 (6.2%)	**2.57 (1.62–4.08)**
Aboriginal/Torres Strait/South Sea Island heritage	42 (4.3%)	49 (5.1%)	1.17 (0.79–1.74)
Born in Australia	907 (91.8%)	879 (90.9%)	0.99 (0.96–1.02)
Mode of arrival			
Aeromedical retrieval	6 (0.6%)	6 (0.6%)	1.05 (0.34–3.22)
Road ambulance	398 (40.0%)	328 (33.8%)	**0.84 (0.75–0.95)**
Police	190 (19.1%)	205 (21.2%)	1.10 (0.93–1.31)
Self, family, friends	516 (51.8%)	573 (59.2%)	**1.15 (1.06–1.24)**
Community MH team/school	13 (1.3%)	23 (2.4%)	1.81 (0.92–3.53)
Service use patterns			
Out‐of‐hours presentation (18:00–07:59)	500 (50.2%)	531 (54.9%)	**1.09 (1.00–1.19)**
High triage urgency (ATS 1 or 2)	219 (22.0%)	235 (24.3%)	1.12 (0.96–1.31)
Visited this ED in last 12 months	357 (35.9%)	431 (44.5%)	**1.24 (1.11–1.38)**
Previous visits^d^	1.0 (1.0–3.0)	2.0 (1.0–4.0)	**1.16 (1.00–1.34)** ^d^
Frequent ED presentations (> 5 per year)	48 (4.8%)	84 (8.7%)	**1.79 (1.28–2.51)**
Seen by ED clinician	874 (92.2%)	885 (93.3%)	1.01 (0.99–1.04)
Median (IQR) wait time for ED clinician (hours)	0.8 (0.3–1.8)	1.2 (0.4–2.6)	**1.49 (1.34–1.65)** ^d^
Seen by mental health clinician	635 (64.2%)	645 (68.2%)	1.05 (0.99–1.11)
Median (IQR) wait time for mental health (hours)	2.5 (1.1–4.6)	3.5 (1.7–6.2)	**1.40 (1.24–1.58)** ^d^
Median (IQR) ED length of stay (hours)	4.7 (3.0–7.6)	5.4 (3.5–10.1)	**1.19 (1.12–1.28)** ^d^
ED short stay unit admission	94 (9.5%)	124 (12.9%)	**1.37 (1.07–1.75)**
Final disposition at discharge			
Admitted/transferred for admission	178 (18.0%)	190 (19.8%)	Reference
Discharged	775 (78.2%)	737 (76.6%)	0.98 (0.93–1.02)
Discharged against medical advice/left without being seen	38 (3.8%)	35 (3.6%)	1.07 (0.70–1.64)
Total length of stay in inpatient unit (hours)	88.8 (25.4–223.2)	114.8 (33.8–232.6)	1.13 (0.84–1.52)^d^

*Note*: Bolded coefficients are significant at the *p* < 0.05 level.

^a^ For categorical variables, percentages (counts) are reported for each year.

^b^ Including transgender boys (*N* = 26) and girls (*N* = 6).

^c^ Gender and sexual diversity—due to low numbers, individuals identifying as transgender or non‐binary and those reporting same‐sex attraction have been combined into a single category. The authors acknowledge that these factors represent different domains of identity and may be associated with different psychosocial and clinical risk profiles.

^d^ For continuous variables, risk ratios of geometric means are reported.

In both years, almost half of all presentations occurred during daytime hours, with a 9% higher likelihood of out‐of‐hours presentations in 2021 (Table 1). Over half arrived on their own or with friends and/or family members in both years, with a higher likelihood of this arrival mode and a lower likelihood of arrival via ambulance in 2021.

Compared to 2019, Median LOS in the ED increased in 2021, and the likelihood of admission to the ED short stay unit was 37% higher. Patients waited longer on average to be seen by ED clinicians in 2021 and time to be seen by a mental health clinician increased (Table 1).

In 2021 participants were 1.24 times more likely to have presented to the same ED in the previous 12 months, and the number of previous presentations per patient was 16% higher (Table 1). We observed no changes in the likelihood of patients being discharged or leaving without being seen/against medical advice. Similarly, there was no change in length of inpatient stay.

### 3.2 Psychosocial Factors

**FIGURE 2:**
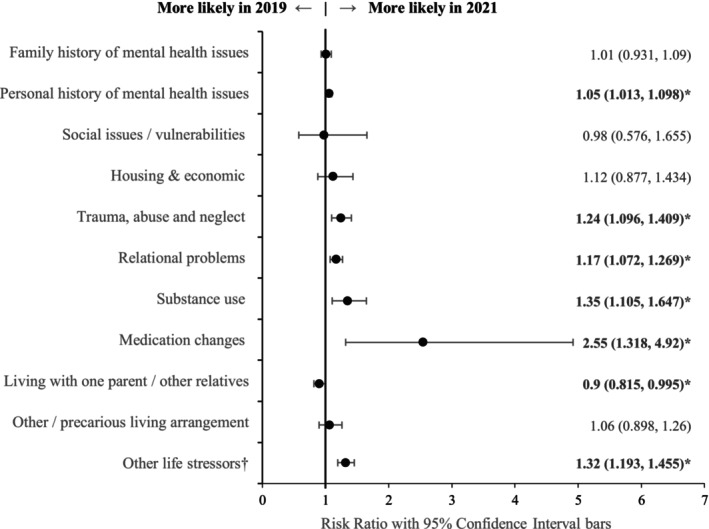
Risk ratio coefficient plot of psychosocial factors associated with presentations in 2021, compared to 2019. ^†^Includes schoolwork/exams, school refusal/disengagement, sleep issues, gaming/social media, travel, relocation, and appearance and health‐related stressors. *Bolded coefficients are significant at the *p* < 0.05 level.

Figure 2 shows the psychosocial factors associated with presentations in 2019 and 2021. In 2021, young people presenting to the ED were more likely to be using substances; to present with relational issues (e.g., bullying, romantic relationship breakdown, and family discord); to have documented past or present trauma, neglect, and abuse; and to present with other life stressors (e.g., school pressures, change in environment, appearance or health‐related stress, and sleeping difficulties). Medication changes were more likely to be a precipitant for presentations in 2021.

Young people presenting to the ED in 2021 were slightly less likely to be living in single‐parent households or with other relatives than to be living with both parents compared to those presenting in 2019. The proportion living in either foster care, residential care, temporary/crisis accommodation, or no fixed address (other/precarious living arrangements) remained stable.

Notably, there was an overall increase in documentation of psychosocial factors associated with presentations in 2021 (RR = 1.13; 95% CI [1.09–1.18]).

### 3.3 Diagnoses and Diagnostic Impressions

**FIGURE 3:**
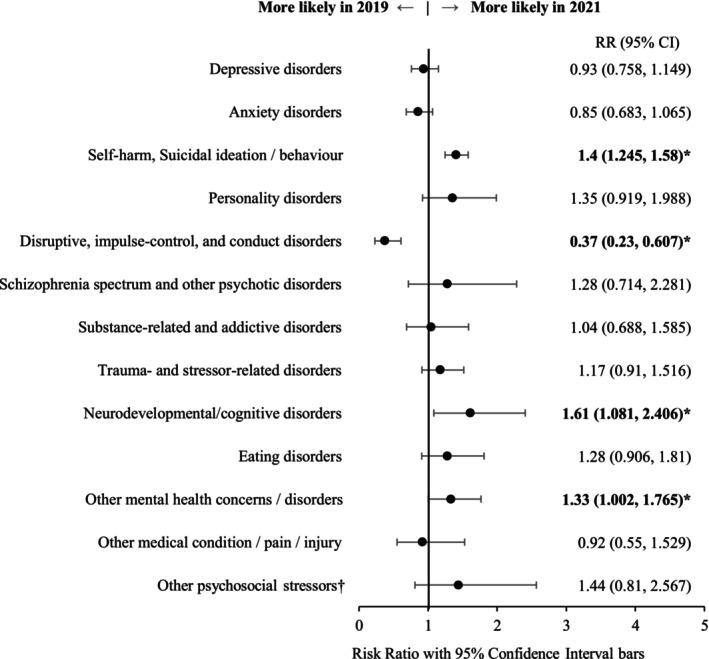
Risk ratio coefficient plot of discharge diagnoses associated with presentations in 2021, compared to 2019. ^†^Includes child–parent relational problems, family conflict and domestic violence, child maltreatment, and other/unspecified social or psychosocial stressors.

Figure 3 provides an overview of the relative risk of documentation of one or more diagnoses/diagnostic impressions in 2021 compared to 2019. We observed significant increases in the likelihood of presentations with documented self‐harm and suicidal ideation and behaviour, neurodevelopmental and neurocognitive disorders, and other mental health concerns/disorders (including obsessive compulsive disorder, bipolar disorder, undefined aggression and behavioural issues, and other mental health concerns not further specified). We found a decreased likelihood of presentations with documented disruptive, impulse control, and conduct disorders in 2021.

## 4 Discussion

The aim of this multisite retrospective cohort study was to compare the profiles of 6–17‐year‐old children presenting to hospital EDs in Australia for mental health‐related reasons in 2019, before the onset of the COVID‐19 pandemic, with those in 2021—the second year of the pandemic. We identified changes in the demographic, psychosocial, and clinical characteristics of those presenting in 2021. Many of our findings align with earlier research, highlighting an increased likelihood of presentations amongst girls, adolescents, and young people presenting with intentional self‐harm, suicidal thoughts and behaviours [11, 12, 13, 14, 23]. Our study extends previous findings, identifying an increase in gender and/or sexual diversity (GSD) of young people presenting to ED; median wait times and length of stay in ED; neurodevelopmental and neurocognitive diagnoses; and reports of psychosocial stressors.

The finding of increased documentation of GSD in 2021 aligns with reports of heightened mental health impacts of the pandemic on GSD young people [24]. However, some caution must be exercised in interpreting these results. GSD data was not routinely or systematically collected at participating sites in 2019 and 2021. Furthermore, GSD may be under‐disclosed due to stigma and negative healthcare experiences [25]. Notably, only a small proportion of patients were identified as transgender (*N* = 32, < 2%) and these patients did not account for the significant increase in girls presenting with mental health concerns. In fact, 81% of transgender young people in this sample were transgender boys (*N* = 26), indicating that increases for children and adolescents assigned female sex at birth would be higher than the gender difference reported. It is possible that changes between 2019 and 2021 resulted from increased awareness and reduced stigma within hospitals, rather than from pandemic impacts. Recent years have seen increased focus on this issue, with the Victorian Government making documentation of preferred gender mandatory in Victorian EDs from 2024 [26].

Changes in time to being seen and LOS are notable, given the wait times already experienced by mental health patients [27]. Aligned with our findings, AIHW reported significantly higher wait times for mental health patients over 2020–2022 [28]. It is possible that these increased wait times related to greater complexity of cases or difficulties in arranging timely and accessible community follow‐up. Whilst this cannot be verified by the descriptive results of the current study, it is partially supported by increased reporting of psychosocial factors and a higher likelihood of being referred to ED short stay. However, this study did not identify a change in presentations triaged as high urgency, nor was there an increase in presentations by ambulance and/or police. It is also likely that changes in the triaging and staffing practices within EDs associated with the pandemic contributed to increased wait times and LOS [10]. For example, staff may have had to engage in time‐consuming practices to prevent iatrogenic spread of COVID‐19 and other illnesses. Following the disruptions associated with the COVID‐19 pandemic, reducing wait times for mental health patients remains a priority, one that will require improvements not only within EDs, but within the referral and treatment pathways available to ED staff for mental health patients [27].

Previous studies found no significant increases in child and adolescent neurodevelopmental disorder presentations in 2020 [13], and a decline amongst young adults [23]. In 2021, we found increases associated with neurodevelopmental and neurocognitive disorders. This may be a result of gradual increases in diagnoses within the community, with data from the Australian Government Pharmaceutical Benefits Scheme (PBS) showing a steady increase in annual spend on psychostimulants and nootropics (typically used in the treatment of ADHD) from 2017/18 to 2021/22 [29]. However, evidence of sub‐optimal access to services and behavioural difficulties in this cohort during and following restrictions [30] suggest that COVID‐19‐related factors may also be implicated. It is possible that the change may be explained by the concurrent decrease in disruptive, impulse‐control, and conduct disorder presentations through a shift in diagnostic practices, given the overlap between these externalising disorders [31]. Similarly, sex could be a factor, given the increased focus on female ADHD and neurodiversity and acknowledgement of historic underdiagnosis for female patients [32]. However, further research is needed to determine the nature and extent of pandemic‐related disruption to the developmental trajectories of young people with neurodevelopmental disorders.

Increases in presentations attributed to medication‐related changes (e.g., side‐effects from commencement/cessation of medication and medication non‐adherence) suggest a possible pandemic impact on prescription and usage of medications. However, these findings are limited by our exploratory design. Despite increased Australian government spending on mental health‐related medications in 2020/2021 [29], there is conflicting evidence on pandemic impacts on prescription and adherence to psychotropic medicine. A study of general practitioner prescribing practices found reductions in new prescriptions associated with telehealth utilisation during lockdowns [33], contradicting possible increases associated with a shift from face‐to‐face to telehealth treatment. Further research can determine the extent and nature of the COVID‐19 pandemic impact on medication prescriptions and adherence.

### 4.1 Strengths and Limitations

By sampling across diverse hospital sites, our methodology allows examination of broader trends. Our study utilised a more in‐depth data collection approach than previous studies, enabling a richer profile of presenting young people to emerge.

There are also limitations. Use of a single year pre‐ and post‐pandemic means we cannot determine whether these changes were expected based on prior years. However, our findings are broadly similar to those of studies which modelled changes over time [11, 13]. Hospitals were recruited via convenience sampling and may not be representative at the national/state level. Almost all sites were metropolitan, and most were based in Victoria, with only two interstate hospitals participating. This may skew results towards the Victorian experience, particularly that of metropolitan Melbourne. However, given the stringency of restrictions in Victoria [3], this may have increased the likelihood of detecting the impact of the COVID‐19 pandemic. A sensitivity analysis was conducted excluding non‐Victorian sites (Supporting Information A), which found a slight increase in the strength of some differences (except arrival by ambulance and admittance to ED short stay, which were no longer significant, Table S5), but minimal changes overall (Table S5, Figures S1 and S2). The reduced likelihood of missing data in 2021 indicates a possible bias, either in data collection or in ED recording practices between years. Diagnostic and psychosocial categories were created by combining original questionnaire items with those that emerged from the open‐ended ‘other’ option provided to abstractors, the latter of which has a lower likelihood of being systematically collected. These limitations are common within medical record cohort studies and are balanced by the reduced risk of bias associated with data collected for non‐research purposes [18]. Finally, it remains possible that the use of mental health diagnoses to describe child and adolescent behavioural and emotional problems may have unintended consequences, including stigma and risk of pathologisation and medicalisation of transient behaviours associated with social and environmental issues [31]. Controversy surrounds the use of mental health diagnostic labels for adolescent populations, due to the ongoing development of personality structures throughout adolescence and early adulthood and the heterogeneity of diagnoses across the life course [31]. In this study, we included psychosocial factors where available for a more holistic view of the patients and the elements contributing to their presentation. However, future research could examine psychosocial and biological determinants of mental health presentations and the supports available in addressing these, with less emphasis on diagnoses.

## 5 Conclusions

Our study identified changes in the demographic, psychosocial and clinical profiles of young people presenting to the ED for mental health assessment in 2021, compared to 2019. We found that young girls, gender and sexually diverse (GSD) young people, and those with associated psychosocial factors, were at increased risk of presenting in 2021. The likelihood of presentations related to intentional self‐harm and neurodevelopmental and neurocognitive disorders increased, while disruptive, impulse‐control, and conduct disorder presentations were less likely. Ongoing challenges associated with wait times in the ED may have been exacerbated by the pandemic. Consideration should be given to (1) the strengthening of services aimed at diverting mental health patients from EDs and ensuring timely and responsive care, and (2) the development of future pandemic mental health‐response guidelines to ensure adaptability of services to meet the needs of young Australians, particularly girls and GSD young people.

## Ethics Statement

The Monash Health Human Research Ethics Committee granted approval for this study (protocol no. RES‐19‐0000‐896A).

## Consent

Informed consent was waived due to the retrospective observational nature of electronic medical record system data. This study was carried out in accordance with the relevant guidelines and regulations.

## Conflicts of Interest

The authors declare no conflicts of interest.

## Supporting information


Supplementary File A.

Table S1.

Table S2.

Table S3.

Table S4.
