# Supplementary material for: Paediatric Emergency Department Mental Health and Behavioural Presentations in Australia Before and After the Onset of the COVID‐19 Pandemic: Retrospective Observational Study
Source: J Paediatr Child Health. 2025 Mar 31;61(6):910–8. doi: 10.1111/jpc.70046 (PMC12128717; doi:10.1111/jpc.70046)
Supplement: Supplementary file 1 — Supplementary File A. [file JPC-61-910-s005.docx]

Supplementary File A: Sensitivity analysis with Victorian sites only

When non-Victorian sites were removed, the reduced risk of arrival by Ambulance and increased risk of an ED short-stay admission observed in the primary analysis were no longer statistically significant (see risk ratios highlighted in grey in **Table S5**). All other comparisons were unchanged.

Table S5. Comparison of demographic, service use, and discharge disposition characteristics of child and youth mental health ED presentations in 2019 and 2021

|  | 2019 vs 2021 RR (95% CI) |
| --- | --- |
| Demographic profile | |
| Age group (years) ^†^ |  |
| Children (6–11) | Reference |
| Youth (12–17) | 1.04 (1.01–1.07) |
| Girls^⸸^ | 1.10 (1.03–1.18) |
| Gender and Sexual Diversity^‡^ | 2.53 (1.56–4.10) |
| Aboriginal/Torres Strait/South Sea Island heritage | 1.51 (0.84–2.70) |
| Born in Australia | 0.98 (0.95–1.01) |
| Mode of arrival | |
| Aeromedical retrieval | 0.64 (0.16–2.63) |
| Road ambulance | 0.90 (0.79–1.02) |
| Police | 1.15 (0.94–1.40) |
| Self, family, friends | 1.12 (1.03–1.22) |
| Community MH team/School | 1.57 (0.79–3.13) |
| Service use patterns | |
| Out-of-hours presentation (18:00–07:59) | 1.08 (0.99–1.19) |
| High triage urgency (ATS 1 or 2) | 1.16 (0.98–1.37) |
| Visited this ED in last 12 months | 1.27 (1.13–1.44) |
| Previous visits^§^ | 1.26 (1.07–1.48)^§^ |
| Frequent ED presentations (>5 per year) | 2.09 (1.41–3.09) |
| Seen by ED clinician | 1.01 (0.98–1.04) |
| Median (IQR) wait time for ED clinician (hours) | 1.70 (1.50–1.92)^§^ |
| Seen by mental health clinician | 1.05 (0.99–1.11) |
| Median (IQR) wait time for mental health (hours) | 1.47 (1.29–1.68)^§^ |
| Median (IQR) ED length of stay (hours) | 1.22 (1.13–1.32)^§^ |
| ED short stay unit admission | 1.20 (0.88–1.62) |
| Final disposition at discharge | |
| Admitted/transferred for admission | Reference |
| Discharged | 0.99 (0.94–1.04) |
| Discharged against medical advice/Left without being seen | 1.08 (0.66–1.74) |
| Total length of stay in inpatient unit (hours)^b^ | 1.19 (0.86–1.64)^§^ |

† For categorical variables, percentages (counts) are reported for each year

⸸ Including transgender girls (N = 6)
‡ Gender and sexual diversity - due to low numbers, GSD has been combined into a single category. Individuals identified as transgender are represented in this category. The authors acknowledge that these factors represent different domains of identity and may be associated with different psychosocial and clinical risk profiles
§ For continuous variables, risk ratios of geometric means are reported

As can be seen in **Figure S1**, removal of non-Victorian sites did not alter the statistically significance of findings regarding changes in psychosocial factors associated with presentations in 2021 compared to 2019, and the risk ratios and confidence intervals remained largely unchanged.

More likely in 2019 | More likely in 2021

Figure S1. Risk ratio coefficient plot of psychosocial factors associated with presentations in 2021, compared to 2019
† Includes schoolwork/exams, school refusal/disengagement, sleep issues, gaming/social media, travel, relocation, and appearance and health related stressors
* Bolded coefficients are significant at the p < 0.05 level

Similarly, there was limited change observed in the risk ratios of discharge diagnoses in 2021 compared to 2019 (**Figure S2)**. When non-Victorian sites were removed, the change in the diagnostic grouping of “Other mental health concerns / disorders” was no longer significant.


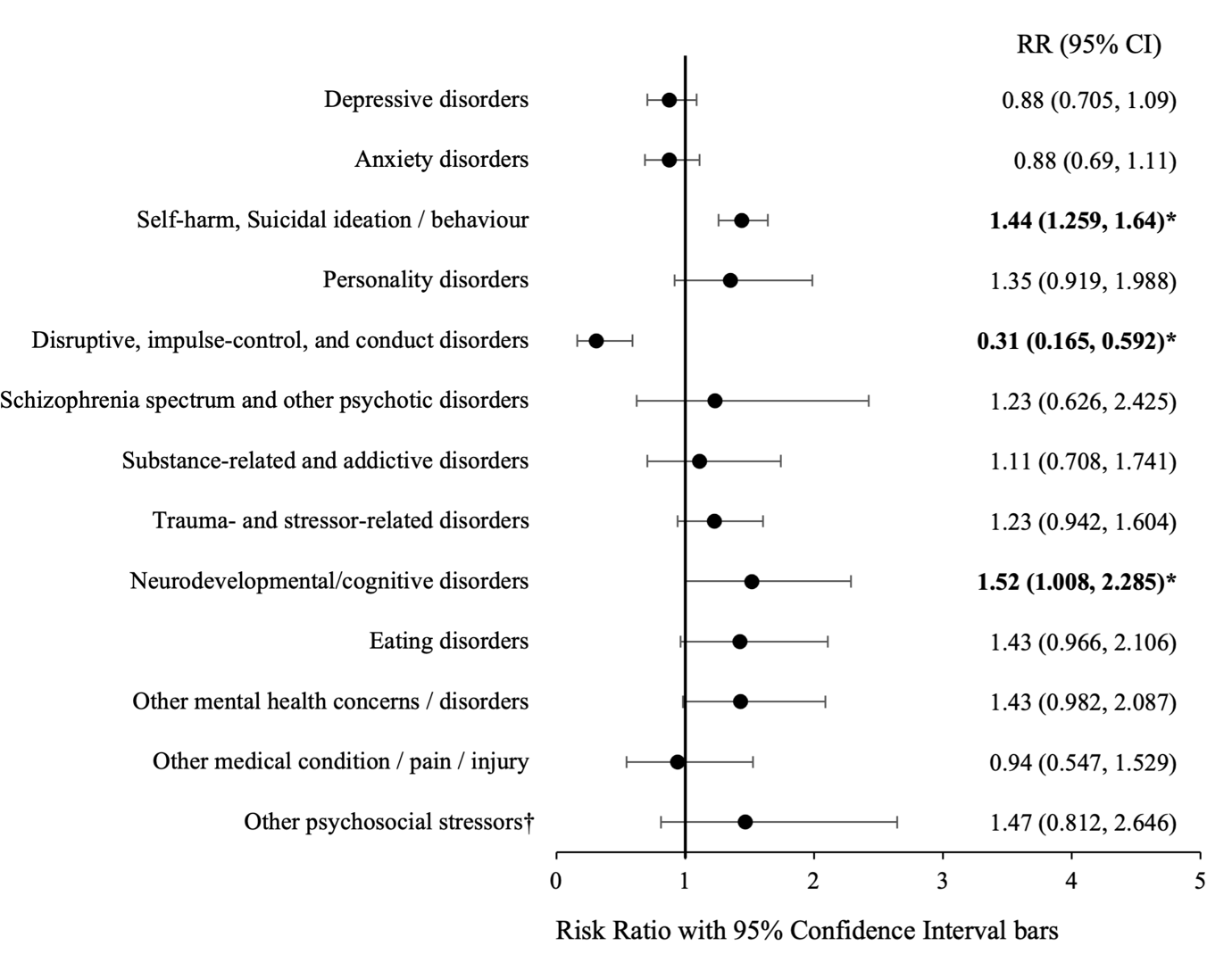


More likely in 2019 | More likely in 2021

Figure S2. Risk ratio coefficient plot of discharge diagnoses associated with presentations in 2021, compared to 2019
† Includes child-parent relational problems, family conflict and domestic violence, child maltreatment, and other/unspecified social or psychosocial stressors
