# Supplementary material for: Paediatric Emergency Department Mental Health and Behavioural Presentations in Australia Before and After the Onset of the COVID‐19 Pandemic: Retrospective Observational Study
Source: J Paediatr Child Health. 2025 Mar 31;61(6):910–8. doi: 10.1111/jpc.70046 (PMC12128717; doi:10.1111/jpc.70046)
Supplement: Supplementary file 3 — Table S2. [file JPC-61-910-s002.docx]

**Table S2.** List of DSM-5 categories with subcategories summarising the diagnostic codes and impressions extracted from the medical records of participating patients.

| **DSM-5 Categories with the categories coded to them from the medical records** |
| --- |
| **Anxiety disorders** |
| Anxiety |
| Selective mutism |
| Social anxiety |
| Panic attack / panic disorder |
| **Depressive disorders** |
| Depressive symptoms |
| Postpartum depression |
| Dysthymia |
| Premenstrual dysphoric disorder |
| Depressive disorder / episode |
| **Disruptive, impulse-control, and conduct disorders** |
| Behavioural problems, child / School refusal / Conduct disorder unspecified |
| Conduct disorder |
| Childhood and adolescence behavioural not otherwise specified |
| Oppositional defiant disorder |
| **Feeding and eating disorders** |
| Disordered eating behaviour |
| Emerging eating disorder |
| Anorexia nervosa |
| Bulimia |
| Eating disorder not otherwise specified |
| Avoidant / restrictive eating disorder |
| **Neurodevelopmental / neurocognitive disorders** |
| Neurodevelopmental disorders (FASD, ASD, ADHD)^1^ |
| Neurocognitive symptoms / disorders (e.g., tic disorder, concussion) |
| Intellectual and learning difficulties / disability |
| **Personality disorders** |
| Axis 2 personality structure / traits |
| Cluster B personality disorder |
| Cluster B Personality Traits |
| Emerging borderline personality disorder |
| Personality disorder unspecified |
| **Schizophrenia spectrum and other psychotic disorders** |
| Psychotic episode |
| Psychotic symptoms |
| Psychosis |
| Schizophrenia |
| Drug-induced psychosis / psychotic symptoms |
| Hallucinations / paranoia / delusions |
| **Substance-related and addictive disorders** |
| Alcohol / drug abuse |
| Withdrawal due to multiple / unspecified drugs or substances |
| Drug misuse/ dependence |
| Mental & behavioural disorder due to harmful use of alcohol / other substances |
| **Trauma- and stressor-related disorders** |
| Adjustment reaction/disorder |
| Complex trauma |
| Complex post-traumatic stress disorder |
| Post-traumatic stress disorder |
| Reactive attachment disorder |
| Post-traumatic stress disorder |
| Situational / emotional crisis, acute stress reaction |
| **Self-harm, suicidal ideation / behaviour** |
| Parasuicidal behaviour |
| Self inflicted lacerations |
| Self-harm |
| Self-harm thoughts |
| Suicide attempt (overdose) |
| Suicide attempt without injury / ideation |
| Suicide risk |
| Suicide attempt |
| Non-suicidal self-harm |
| Ingestion/ overdose (where self-harm intent specified) |
| **Psychosocial stressors** |
| Psychosocial stressors not otherwise specified |
| Domestic violence / trauma / abuse |
| Child, parent and family relationship conflict / difficulties / disorder |
| **Other behavioural or mental health concern / disorder** |
| Acute behavioural disturbance |
| Attention-deficit hyperactivity disorder / autism symptoms |
| Bipolar affective disorder |
| Body dysmorphia |
| Emotional dysregulation |
| General psychiatric exam |
| Insomnia |
| Somatic symptom and related disorders |
| Gender dysphoria / gender identity disturbance |
| Anger / aggression issues |
| Behavioural / mental health problems not otherwise specified |
| Disruptive behaviour / agitation |
| Obsessive compulsive disorder / symptoms |
| Odd / abnormal / altered behaviour |
| **Other medical / pain / injury** |
| Acute sinusitis |
| Arrhythmia |
| Asthma |
| Disorder of electrolytes |
| Dysphagia |
| Fracture |
| General physical examination |
| hyperemisis |
| Hyperglycaemia |
| Localised cutaneous vasculitis |
| Medication changes |
| Postural hypotension |
| Syncope |
| Epilepsy / seizure-like activity |
| Superficial injury of ankle / foot / neck / wrist / hand / arm |
| Burn / corrosion |
| Chest pain |
| Constipation |
| Ear nose and throat pain |

1. Foetal alcohol spectrum disorder (FASD), autism spectrum disorder (ASD), attention deficit hyperactivity disorder (ADHD)
