# Supplementary material for: Paediatric Emergency Department Mental Health and Behavioural Presentations in Australia Before and After the Onset of the COVID‐19 Pandemic: Retrospective Observational Study
Source: J Paediatr Child Health. 2025 Mar 31;61(6):910–8. doi: 10.1111/jpc.70046 (PMC12128717; doi:10.1111/jpc.70046)
Supplement: Supplementary file 4 — Table S3. [file JPC-61-910-s001.docx]

**Table S3.** Total number of visits/patients and final recruitment numbers at each participating site

|  |  | **2019** | | |  | | **2021** | | |
| --- | --- | --- | --- | --- | --- | --- | --- | --- | --- |
|  | **Site characteristics** | **Total visits** | **Unique patients** | **Included patients** |  | **Total visits** | | **Unique patients** | **Included patients** |
| Site 1 | Paediatric tertiary referral hospital, VIC | 1,183 | 862 | 107 |  | 1,842 | | 1,090 | 100 |
| Site 2 | Mixed non-tertiary hospital, VIC | 290 | 238 | 101 |  | 266 | | 171 | 100 |
| Site 3 | Mixed non-tertiary hospital, VIC | 606 | 483 | 104 |  | 522 | | 402 | 106 |
| Site 4 | Mixed tertiary hospital, VIC | 841 | 515 | 101 |  | 1,314 | | 645 | 95 |
| Site 5 | Mixed non-tertiary hospital, VIC | 342 | 283 | 100 |  | 442 | | 339 | 99 |
| Site 6 | Mixed non-tertiary hospital, VIC | 107 | 82 | 82 |  | 97 | | 81 | 68 |
| Site 7 | Paediatric tertiary referral hospital, VIC | 1,964 | 1,314 | 100 |  | 3,079 | | 1,784 | 100 |
| Site 8 | Mixed non-tertiary hospital, VIC | 577 | 403 | 100 |  | 642 | | 462 | 100 |
| Site 9 | Mixed tertiary hospital, TAS | 405 | 252 | 101 |  | 557 | | 299 | 100 |
| Site 10 | Mixed non-tertiary hospital, QLD | 951 | 636 | 100 |  | 1,022 | | 703 | 100 |
| **Total** | **All sites** | **7266** | **5067** | **996** |  | **9783** | | **5976** | **968** |
