# Supplementary material for: Paediatric Emergency Department Mental Health and Behavioural Presentations in Australia Before and After the Onset of the COVID‐19 Pandemic: Retrospective Observational Study
Source: J Paediatr Child Health. 2025 Mar 31;61(6):910–8. doi: 10.1111/jpc.70046 (PMC12128717; doi:10.1111/jpc.70046)
Supplement: Supplementary file 5 — Table S4. [file JPC-61-910-s004.docx]

**Table S4.** Description of Australasian Triage Scale (ATS) Categories

| ATS Category | Description of Category | Response |
| --- | --- | --- |
| Category 1 | Immediately Life-Threatening | Immediate simultaneous assessment and treatment |
| Category 2 | Imminently life-threatening | Assessment and treatment within 10 minutes |
| Category 3 | Potentially Life-Threatening | Assessment and treatment start within 30 mins |
| Category 4 | Potentially serious | Assessment and treatment start within 60 mins |
| Category 5 | Less Urgent | Assessment and treatment start within 120 minutes |
